# Metabolic Profiling of *Dendrobium officinale* in Response to Precursors and Methyl Jasmonate

**DOI:** 10.3390/ijms19030728

**Published:** 2018-03-03

**Authors:** Chunyan Jiao, Cheng Song, Siyan Zheng, Yingpeng Zhu, Qing Jin, Yongping Cai, Yi Lin

**Affiliations:** School of Life Sciences, Anhui Agricultural University, Hefei 230036, China; 15212426671@163.com (C.J.); laniao812329218@163.com (C.S.); 13866103668@163.com (S.Z.); 13856983945@163.com (Y.Z.)

**Keywords:** *Dendrobium officinale*, metabolomics, alkaloids, tryptophan, secologanin, methyl jasmonate

## Abstract

Alkaloids are the main active ingredients in the medicinal plant *Dendrobium officinale*. Based on the published genomic and transcriptomic data, a proposed terpenoid indole alkaloid (TIA) biosynthesis pathway may be present in *D. officinale*. In this study, protocorm-like bodies (PLBs) with a high-yielding production of alkaloids were obtained by the optimization of tryptophan, secologanin and methyl jasmonate (MeJA) treatment. The results showed that the total alkaloid content was 2.05 times greater than that of the control group when the PLBs were fed with 9 µM tryptophan, 6 µM secologanin and 100 µM MeJA after 36 days. HPLC analysis showed that strictosidine synthase (STR) activity also increased in the treated plants. A total of 78 metabolites were identified using gas chromatography-mass spectrometry (GC-MS) in combination with liquid chromatography-mass spectrometry (LC-MS) methods; 29 differential metabolites were identified according to the multivariate statistical analysis. Among them, carapanaubine, a kind of TIA, exhibited *dramatically* increased levels. In addition, a possible underlying process of the metabolic flux from related metabolism to the TIA biosynthetic pathway was enhanced. These results provide a comprehensive view of the metabolic changes related to alkaloid biosynthesis, especially TIA biosynthesis, in response to tryptophan, secologanin and MeJA treatment.

## 1. Introduction

Plants of the *Dendrobium* genus, especially *Dendrobium officinale* Kimura et Migo, are traditional Chinese medicinal herbs used for maintaining tonicity of the stomach and promoting body fluid production [1]. The major active ingredients of *D. officinale* are polysaccharides, alkaloids, phenols, terpenes, and flavonoids as well as several trace mineral elements [2,3,4,5]. 

Previous studies have focused on characterizing alkaloid components in *Dendrobium* plants. The total alkaloid content of *D. officinale* has been measured to be 0.02%; although this content is lower than that of *Dendrobium nobile*, *D. officinale* has been demonstrated to produce high-quality alkaloids [6]. Ever since the genome and transcriptome of *D. officinale* were sequenced, results have shown that *D. officinale* may contain terpenoid indole alkaloids (TIAs) [3,7,8,9]. TIAs are mainly found in Apocynaceae family plants, such as *Catharanthus roseus*, a model medicinal plant for the study of TIA biosynthesis. A common precursor for all TIAs is strictosidine, which is assembled from two intermediates: tryptamine and secologanin. The former is synthesized from the indole pathway, while the latter is derived from the terpene biosynthetic pathway; both intermediates then combine with each other by strictosidine synthase (STR) to form strictosidine [10,11].

Elicitation and precursor feeding have been proven to be two effective methods to increase the accumulation of secondary metabolites [12]. Methyl jasmonate (MeJA) has been identified as a signaling molecule that induces gene expression and elicits secondary metabolic pathways in plant cells [13]. The effects of MeJA on the production of bioactive compounds (alkaloids, polysaccharides, and flavonoids) in *D. officinale* protocorm-like bodies has been reported [14,15]. In addition, several attempts to increase the production of TIAs by precursor feeding have been performed, such as the production of reserpine from *Rauvolfia serpentina* fed with tryptamine and the production of ajmalicine, vindoline and catharanthine from *C. roseus* fed with tryptophan, tryptamine, secologanin and loganin [16,17]. Some studies have also shown that it is more effective to promote the accumulation of secondary metabolites by the combination of elicitors and precursors [12]. Therefore, it is feasible to increase total alkaloid contents, especially TIAs, by the combination of TIA precursors and MeJA treatment.

To further investigate the metabolic changes under TIA precursors and MeJA treatment, we employed a metabolomic approach. The combination of gas chromatography-mass spectrometry (GC-MS) and liquid chromatography-mass spectrometry (LC-MS) has been proven to be a powerful tool for unraveling primary and secondary metabolite fluctuations and the comprehensive metabolite network flux in response to environmental factors [18]. For example, GC-MS and LC-MS were used to reveal differential responses in the flavonoid, phenylpropanoid and terpene pathways after MeJA treatment in *Centella asiatica* [19]. Significantly different metabolic profiling between *C. roseus* and *Vinca minor* was characterized using GC-MS and LC-MS methods [20]. Recently, metabolomic characterization based on non-targeted GC-MS of medicinal *Dendrobium* plants was also reported [21,22].

In this study, protocorm-like bodies of *D. officinale* were used as raw materials. TIA precursors (tryptophan and secologanin) and exogenous elicitors (MeJA) were used to induce alkaloid biosynthesis. GC-MS and LC-MS coupled with multivariate data analyses were applied in combination to detect the metabolic changes in primary and secondary metabolism, particularly in TIA biosynthesis. We attempted to explore not only the alkaloid types in *D. officinale* but also the TIA biosynthesis in response to tryptophan, secologanin and MeJA treatments.

## 2. Results

### 2.1. Effects of MeJA and Its Combination with TIA Precursors on D. officinale PLB Biomass, Alkaloid Content and STR Activity

The treatment of TIA precursors (tryptophan and secologanin) and MeJA exhibited marked effects on biomass and total alkaloid content accumulation during *D. officinale* PLB cultures. The biomass and total alkaloid content were determined after 12, 24, 36 and 48 days. As shown from Tables S1 and S2, the fresh weight and dry weight of PLBs both showed a downward trend with all combinations of tryptophan, secologanin and MeJA treatment, that is, PLB growth was slightly inhibited. 

Table S3 shows the effects of TIA precursors and MeJA treatment on the total alkaloid accumulation. The total alkaloid content continuously increased and peaked on day 48 during the whole culture cycle. However, the dry weight of PLBs during this period dramatically decreased (Table S2), and the production of alkaloids also decreased. Moreover, the maximum total alkaloid content (472.33 ± 3.79 µg/g DW) was achieved by feeding with 9 µM tryptophan, 6 µM secologanin and 100 µM MeJA after 36 days (No. 8); this content was almost 2.05 times than that of the control group (Table S3).

Based on the above results, the total alkaloid contents of the different combinations of accumulations of tryptophan, secologanin and MeJA concentrations after 36 days were selected to conduct orthogonal analyses. The results of the orthogonal experiment are listed in Table 1. *K*_1_, *K*_2_ and *K*_3_ were the average values of the results at each level in the orthogonal method experiment, and the optimal level of each variable can be determined by comparing the value of *K*_i_ (*i* = 1, 2, 3). Experimental results showed that the *K* value by magnitude was *K*_3_ > *K*_2_ > *K*_1_, which illustrated that 9 µM tryptophan played a good role in promoting alkaloid accumulation. Similarly, comparing the *K* values of secologanin and MeJA, *K*_2_ and *K*_1_ were higher than the others, which showed that 6 µM secologanin and 100 µM MeJA were beneficial to the production of alkaloids. Therefore, we obtained a theoretical optimal combination of A3B2C1, which was 9 µM tryptophan, 6 µM secologanin and 100 µM MeJA. The range of *K* can show the effect of a certain factor on the total alkaloid accumulation. From the range analysis results, the order of total alkaloid contents that exhibited effects was ranked as C > B > A, that is, MeJA >secologanin >tryptophan. This finding indicated that the MeJA concentration had the highest effect, while the tryptophan concentration had the lowest effect. From the range analysis, we obtained a theoretical optimal combination of A3B2C1, which was 9 µM tryptophan, 6 µM secologanin and 100 µM MeJA. The results of the analysis of variance are shown in Table S4. The results are listed in Table S5. The average total alkaloid content was 473 μg/g DW. This value is similar to the total alkaloid content in the No. 8 test of the orthogonal experiment, indicating that this process might be relatively reliable and stable. 

**Figure 1:**
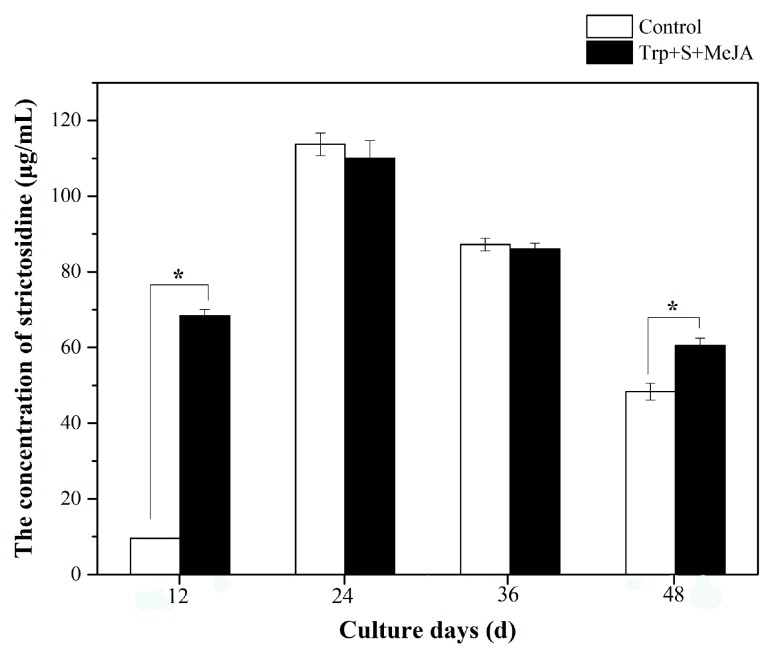
Strictosidine synthase activity during the time course from fed and control samples of *D. officinale* PLBs. Regarding feeding, 9 µM tryptophan (Trp), 6 µM secologanin (S) and 100 µM MeJA applications were applied at day 12, 24, 36 and 48. PLBs: protocorm-like bodies; d: day; *: significant changes from corresponding controls (*p* < 0.01).

The strictosidine synthase (STR) activity profiles under treatment with 9 µM tryptophan, 6 µM secologanin and 100 µM MeJA during the whole culture cycle were also analyzed; the results are shown in Figure 1. The activity of STR was significantly higher than that of the control group (7.23 fold) after 12 days, and the activity peaked in both groups after 24 days. Then, the STR enzyme activity decreased and showed significant differences at day 48. In summary, The TIA precursors (tryptophan and secologanin) and MeJA had positive effects on the activity of the STR enzyme and increased the activity significantly at the beginning of the rapid growth of *D. officinale* PLBs (day 12). Based on the above data, PLBs treated with 9 µM tryptophan, 6 µM secologanin and 100 µM MeJA at day 36 and non-treated samples were collected as materials for GC-MS and LC-MS analyses.

### 2.2. Metabolic Profiling Analysis of D. officinale PLBs Treated with TIA Precursors and MeJA

**Figure 2:**
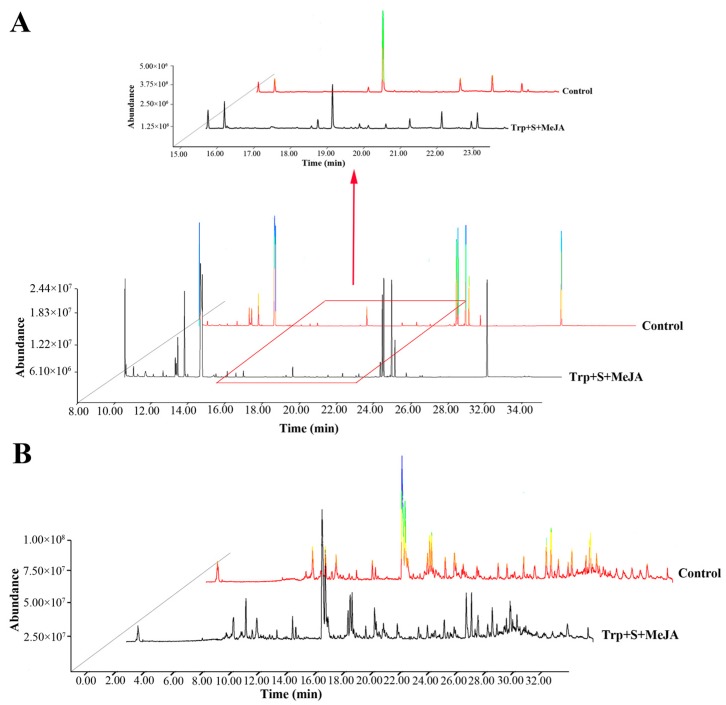
Overlays of total ion chromatograms (TICs) by GC-MS and LC-MS analyses of treated and non-treated groups. (**A**,**B**) are the GC-MS and LC-MS (positive ion mode) chromatograms, respectively.

In this study, metabolites were extracted from the *D. officinale* PLBs by using a combinative treatment of 9 µM tryptophan, 6 µM secologanin and 100 µM MeJA (Trp + S + MeJA) after 36 days and a control group was defined using non-treated samples. Both experimental groups were replicated six times, and the metabolites were analyzed by GC-MS and LC-MS. Clear chromatographic differences were observed between treated and non-treated sample groups, and a total of 78 types of metabolites were identified from all samples (Table S6). Typical TICs are shown in Figure 2 and Figure S1. From the GC-MS data, with reference to the NIST11 database, 45 primary metabolites were putatively identified; these metabolites mainly included amino acids, organic acids, and sugars. Unlike GC-MS, secondary metabolites such as alkaloids, terpenes and flavonoids were detected by LC-MS. Of these, 16 alkaloids were preliminarily identified and were classified into eight categories: TIAs, tropane alkaloids, monoterpene alkaloids, diterpene alkaloids, pyrrolizidine alkaloids, isoquinoline alkaloids, quinolizidine alkaloids and piperidine alkaloids.

**Figure 3:**
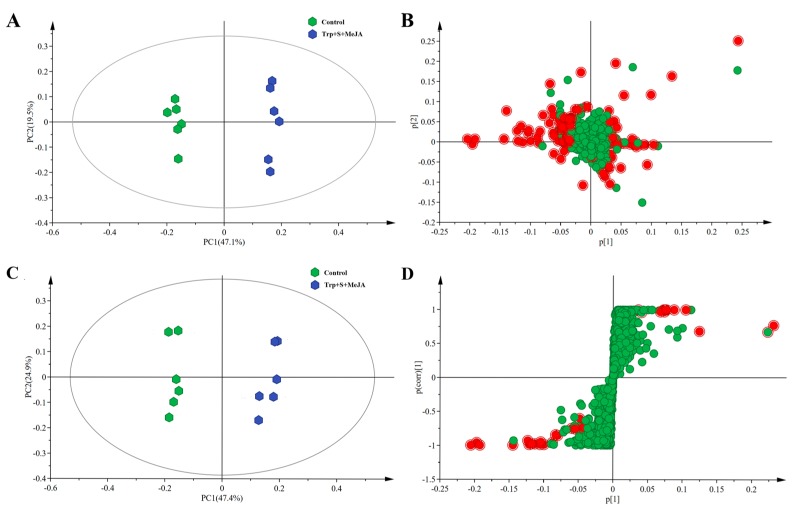
Principle component analysis (PCA) and orthogonal partial least squares-discriminant analysis (OPLS-DA) of metabolites derived from GC-MS and LC-MS data. The data consist of 2232 signals, each corresponding to a unique *m*/*z* and retention combination (Table S6). (**A**,**C**) are the scores plots of PCA and OPLS-DA models, respectively. (**B**,**D**) are the loading plot and S-plot of PCA and OPLS-DA models, respectively. Red dots in loading plot and S-plot means the differential metabolites selected by VIP > 1.5 and *p* < 0.05, and green dots represent other detected signals in Table S6.

For displaying the data structure and differentiation between the treated (Trp + S + MeJA) and non-treated samples (control), we reduced the dimensionality of GC-MS and LC-MS data, and sample groupings were visualized. To better highlight the differences in metabolite profiling of the Trp + S + MeJA-treated and control samples, principal component analysis (PCA) (*R*^2^*X* = 0.666, Q^2^ = 0.559) and orthogonal partial least squares-discriminant analysis (OPLS-DA) (*R*^2^*X* = 0.723, *R*^2^*Y* = 0.994, *Q*^2^ = 0.98) models were applied to the GC-MS and LC-MS data. The PCA score plot (Figure 3A) and loading plot (Figure 3B) of the GC-MS and LC-MS data showed that the first two main principal components (PCs) explained 47.1% and 19.5% of the variance, respectively. In general, the PCA model depicted the sample groupings: all the control samples clustered together and were significantly separated from the treated samples. These results showed that tryptophan, secologanin and MeJA may strongly impact the *D. officinale* PLB metabolome.

The OPLS-DA models (score plot and S-plot) were further applied to observe the predictive variation contributing significantly to the sample separation, with two PCs explaining 47.4% and 24.9% of the GC-MS and LC-MS data, respectively (Figure 3C,D), and showed similar results to those of PCA; clear clustering of *D. officinale* PLBs fed with tryptophan, secologanin and MeJA was observed. The model quality was shown by the values of R^2^X and Q^2^ (Figure S2). The intercept (*R*^2^ and *Q*^2^ when correlation coefficient is zero) which was correlated with the extent of overfitting was rather small (*R*^2^ = 0.602, *Q*^2^ = −0.847) and the model was satisfactory.

**Figure 4:**
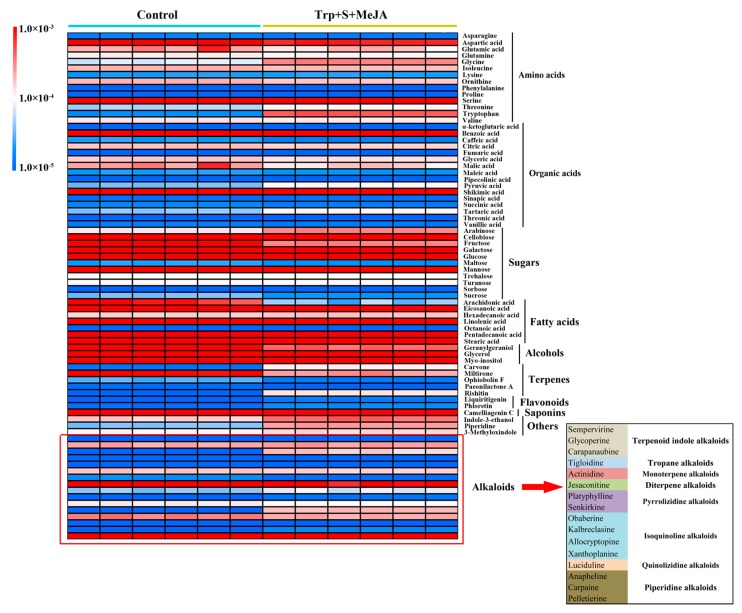
Heatmap analysis of 78 identified metabolites between treated (Trp + S + MeJA) and untreated (control) *D. officinale* PLBs. Normalized peak areas are shown on a color scale (shown at the left) and are provided in Table S6. The lowest ratios are in blue, and the highest ratio in red.

Based on the 78 annotated metabolites, clear dynamic changes were observed between the Trp + S + MeJA-treated and control samples when performing a heat map analysis (Figure 4). The results revealed that alkaloids, terpenes, amino acids and several other compounds changed in the treated groups. Furthermore, to better evaluate the changes in metabolite abundance, a total of 101 discriminating metabolites (from all the detected 2232 signals) were obtained using the OPLS-DA model according to their VIP values (VIP > 1.5) and *p*-values (*p* < 0.05) (Table 2 and Table S7). A VIP > 1.5 means that variables have above average influence on classification and the highest VIP value was 11.53. Next, Student’s t test was further employed to evaluate significant differences (*p* < 0.05) of metabolite candidates and to eliminate variables without significant differences between the treated and control groups. Among the 101 metabolites, 36 metabolites exhibited higher levels in treated samples, while 65 metabolites had decreased levels. In addition, 29 of 101 discriminating metabolites were ascertained as differential metabolites according to the identify check by raw data and the annotation of peaks by searching the databases. The detailed information of these 29 identified metabolites is presented in Table 2. These altered metabolites included four kinds of amino acids, four kinds of alkaloids, five kinds of fatty acids, six kinds of sugars, two kinds of alcohols, two kinds of organic acids, three kinds of terpenes, one kind of saponin and two kinds of other metabolites. Among them, up-regulation of tryptophan (20.65 fold), piperidine (7.24 fold), carvone (8.81 fold), and rishitin (26.74 fold) was observed in the fed PLBs. Carapanaubine, a kind of TIA, *dramatically increased (1928.46 fold) in the treated samples*. In contrast, pelletierine (piperidine alkaloid) and senkirkine (pyrrolizidine alkaloid) had lower abundances in the treated samples; the levels of these metabolites were 0.57- and 0.68-fold lower than those of the control samples, respectively. In addition, the levels of sugars (expect for trehalose), organic acids and some fatty acids sharply decreased. Such phenomena may reflect changes in metabolic pathways, especially the alkaloid biosynthetic pathway.

**Figure 5:**
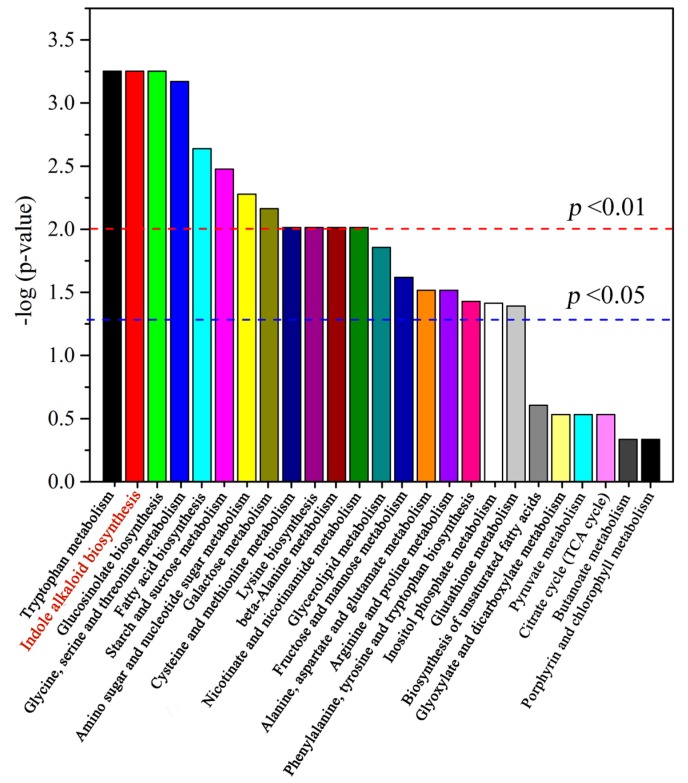
Histogram of pathway analysis. The horizontal line of 1.3 indicates *p* < 0.05; the horizontal line of 1.5 indicates *p* < 0.01.

Subsequently, all 29 differential metabolites were annotated to biological pathways in the KEGG database using MetaboAnylst 3.0 (Table S8) and were assigned to 25 pathways (Figure 5). According to the −log (*p*-value) data, we found that tryptophan metabolism, indole alkaloid biosynthesis and glucosinolate biosynthesis were deeply enriched, indicating that Trp + S + MeJA treatment mainly affects these three metabolic pathways. Other pathways, such as amino sugar and nucleotide sugar metabolism, fatty acid biosynthesis, and galactose metabolism, were also enriched.

### 2.3. Alkaloid Biosynthesis Is Affected by TIA Precursors and MeJA Treatment

**Figure 6:**
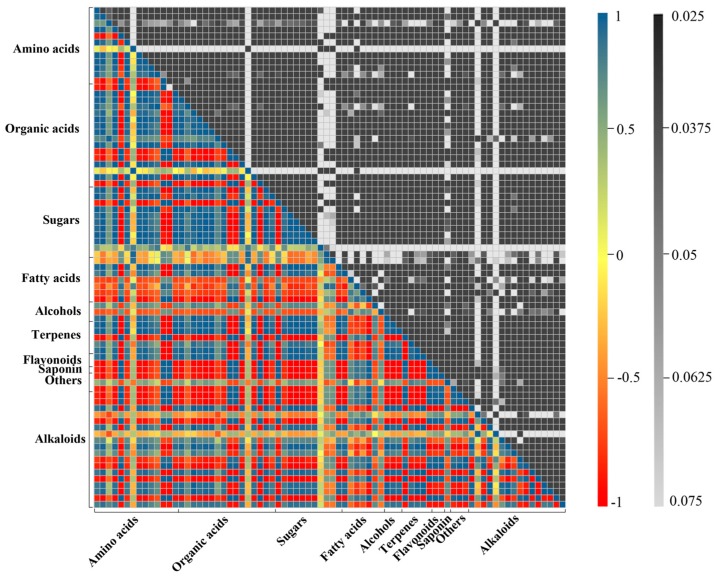
Heat map representing the r coefficients and the corresponding *p*-values for the identified metabolites in *D. officinale* PLBs after feeding with TIAs precursors and MeJA. Correlations including statistical significance of all identified metabolites (listed in Figure 4) are analyzed by using a Pearson correlation coefficient method, and the calculated results are shown in Table S9. In the colored area, rectangles represent Pearson correlation coefficient (*r*) values of metabolites pair (see correlation color key). In the black and white area, rectangles represent the respective *p*-values (see significance color key).

**Figure 7:**
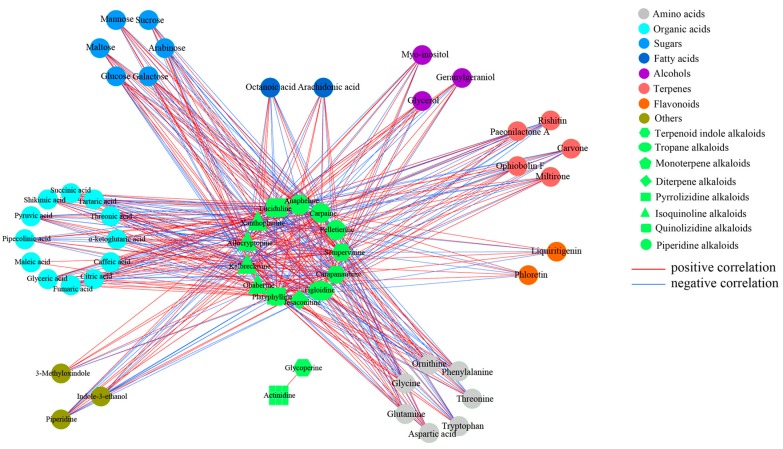
Correlation network based on identified alkaloids and other metabolites with significant correlations. Node colors and shapes represent different types of metabolites. Edges between nodes represent correlation identified as significant at |*r*| > 0.9, *p* < 0.001, where red and blue edges represent positive and negative correlations.

To investigate the fluctuations between alkaloids and other identified metabolites in *D. officinale* PLBs after Trp + S + MeJA treatment, a pairwise correlation analysis was performed by employing the Pearson correlation coefficient method and the correlation network. A total of 3003 correlations were calculated, with r-values ranging from −0.994 for fructose and tryptophan to 1 for galactose and fructose (Figure 6 and Table S9) were obtained. It was found that most of the amino acids except lysine displayed high positive associations with organic acids, sugars and some alkaloids, indicating that these metabolites may share the same biosynthetic pathways and exhibit similar changes in response to Trp + S + MeJA treatment. By contrast, fatty acids correlated negatively with amino acids, organic acids, sugars and some terpenes due to the branches of their biosynthetic pathways. Further screening resulted in identification of 452 significant correlations (246 negative correlations and 206 positive correlations) between alkaloids and other identified metabolites, with *r*^2^ ≥ 0.81 (*r* ≥ 0.9 and *r* ≤ 0.9) and *p* ≤ 0.001. Different types of alkaloids showed different correlations with the related metabolites in response to Trp + S + MeJA treatment (Figure 7). For TIAs, sempervirine showed strong negative correlation with tryptophan (*r* = −0.986, *p* = 4.28 × 10^−8^), while carapanaubine had strong positive correlation with tryptophan (*r* = 0.971, *p* = 6.83 × 10^−7^), though both of them belong to TIAs and tryptophan was used as a common substrate for their biosynthesis. This phenomenon reflects that the downstream components of the TIA pathway of these two alkaloids might be totally different. Carapanaubine was found to have significant negative correlations with sugars, including galactose (*r* = −0.962, *p* = 2.28 × 10^−6^), glucose (−0.960, *p* = 2.71 × 10^−6^), and sucrose (*r* = −0.952, *p* = 6.52 × 10^−6^). The data presented here provide correlative information that may allow the elucidation of the linkage between the biosynthesis pathways of sugars, amino acids, organic acids, terpenes and alkaloids.

**Figure 8:**
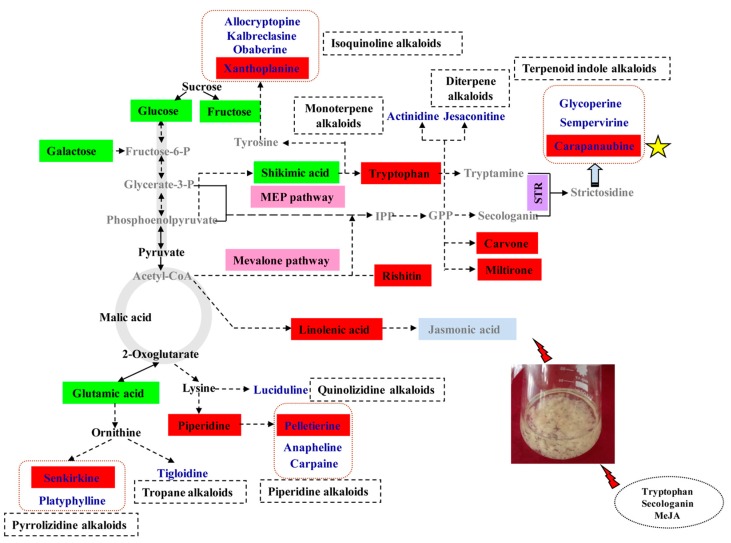
Proposed alkaloid biosynthetic pathway in response to TIA precursors and MeJA. Metabolites in gray indicate not detected in this study; alkaloids are shown in blue characters. Green and red boxes represent the decrease and increase level of metabolites, respectively.

To fully understand the regulation of 16 alkaloid (eight types) metabolic pathways in *D. officinale* PLBs after Trp + S + MeJA treatment, we constructed a metabolic profile with the aid of KEGG (Figure 8). We found that primary metabolism (sugars, amino acids, organic acids, and fatty acids) was closely associated with secondary metabolism (alkaloids and terpenes). The main metabolic changes were concentrated on sugars, alkaloids and terpenes. In particular, shikimic acid and tryptophan were the main metabolic precursors of the TIA biosynthetic pathway, while carapanaubine, sempervirine and glycoperine were the TIAs; this finding further indicates that *D. officinale* contains TIAs and has a TIA biosynthetic pathway. Tryptophan and carapanaubine exhibited significantly increased levels in response to Trp + S + MeJA treatment, although shikimic acid significantly decreased. A similar variation pattern was also observed for xanthoplanine and for some terpenes, such as carvone, rishitin and miltirone (Figure 8). Other types of alkaloids, especially senkirkine and pelletierine, which are derived from ornithine and lysine, respectively, showed a variation pattern that was opposite that of TIAs. It was interesting that metabolites involved in sugar metabolism, such as glucose, fructose and galactose, also showed less abundances under Trp + S + MeJA treatment. Given these observations, we implied that the altered abundances of these metabolites may reflect the enhanced metabolic fluxes in TIA biosynthesis as well as in terpene biosynthesis, whereas the metabolic fluxes in sugars and other types of alkaloid biosynthesis (pyrrolizidine alkaloid and tropane alkaloid metabolism) were strongly decreased, indicating a regulatory cross-interaction between primary and secondary metabolism. In addition, an increased level of linolenic acid may also affect TIA biosynthesis.

## 3. Discussion

### 3.1. Alkaloid Types and Their Putative Biosynthetic Pathways in D. officinale

Alkaloids are the first category of bioactive components extracted and identified from *Dendrobium* plant species [2]. And Tandem MS studies have contributed to the identification and characterization of alkaloids in Dendrobium genus [23]. Thus far, five types of alkaloids with confirmed structures have been reported, namely, sesquiterpene alkaloids, indolizidine alkalioids, pyrrolidine alkaloids, phthalide alkaloids and imidazole alkaloids [2]. However, no single kind of alkaloid has been reported in *D. officinale*. In previous research, unigenes associated with TIA, isoquinoline alkaloid, tropane and piperidine alkaloid pathways were annotated from *D. officinale* transcriptome data [3,7,9]; these results correspond to our metabolomics results (Figure 4). 

The sesquiterpene backbone of dendrobine in *D. nobile* was verified to be involved in the mevalonate (MVA) pathway or the 2-*C*-methyl-d-erythritol 4-phosphate (MEP) pathway [24], suggesting that the terpene biosynthetic pathway may form the terpene backbones of different types of terpenoid alkaloids. Genes such as 1-deoxy-d-xylulose 5-phosphate synthase (*DXS*) and 1-deoxy-d-xylulose-5-phosphate reductoisomerase (*DXR*), which are related to the MEP pathway, as well as hydroxymethylglutaryl-CoA synthase (*HMGS*) and3-hydroxy-3-methyl-glutaryl-coenzyme A reductase (*HMGR*), which are related to the MVA pathway, have been annotated in *D. officinale* [9,25], whereas terpenes such as carvone (a monterpene), miltirone (a diterpene) and rishitin (a sesquiterpene) were identified in our experiments. Though sesquiterpene existed in *D. officinale*, no sesquiterpene-type alkaloid (dendrobine) was detected in our study, indicating that *D. officinale* may not contain it [26]. Moreover, other genes, such as *STR* and tryptophan decarboxylase (*TDC*), of key enzymes involved in the TIA biosynthetic pathway, have also been identified in *D. officinale* [24]. Overall, we confirmed that the terpene backbones of TIAs (sempervirine, glycoperine and carapanaubine), monoterpene alkaloids (actinidine) and diterpene alkaloids (jesaconitine) were all associated with the MVA and MEP pathways. With respect to quinolizidine alkaloids (luciduline) and piperidine alkaloids (anapheline, carpaine and pelletierine), their structures contained piperidine backbones that originated from lysine (Figure 8). The biosynthetic pathway of indolizidine alkaloids in *Dendrobium crepidatum* has already been studied [27]. Other types of alkaloids, including tropane alkaloids [28], pyrrolizidine alkaloids [29], and isoquinoline alkaloids [30] as well as their biosynthetic pathways have been reported in other plants. These above results provide a new reference for studying the different types of alkaloid biosynthetic pathways in *D. officinale*.

### 3.2. Metabolic Changes in Alkaloid Biosynthesis in D. officinale in Response to TIA Precursors and MeJA Treatment

MeJA has been found to switch on the gene expression of different secondary metabolites, leading to dynamic metabolic changes that involve alterations in some steps of the biosynthesis of these metabolites [31,32]. Several studies have been reported in which the activity of STR and the expression of *STR* were both up-regulated by JA or MeJA feeding; both metabolites play a role in activating TIA biosynthesis [33,34,35]. Our results also found that exogenous feeding of MeJA may have a more positive effect on STR activity, while tryptophan and secologanin feeding increased the catalytic efficiency of STR. All the above results may lead to alkaloid accumulation in *D. officinale* PLBs. It is interesting that an increasing level of linolenic acid was also observed in treated *D. officinale* PLBs (Table 2)*.* MeJA is a type of jasmonate (JA), it is synthesized in plants via the octadecanoid pathway and is formed from linolenic acid [36]. Several mRNAs coding for enzymes involved in JA biosynthesis were up-regulated upon JA treatment, and endogenous JA levels increased [37,38]. Our findings showed that an increase in linolenic acid may result in the accumulation of endogenous JA, which could trigger alkaloid biosynthesis in *D. officinale*. 

Metabolite changes were reflected in the flux analysis, which revealed the metabolic shifts [39]. According to the GC-MS and LC-MS data, 29 metabolites changed significantly in response to TIA precursors (tryptophan and secologanin) and MeJA treatment. These metabolites were annotated to different metabolic pathways, including alkaloid, terpene and sugar metabolism. Our results showed a decrease in pelletierine, senkirkine, glucose, fructose and galactose, and an increase in tryptophan and carapanaubine, indicating that the flux change from related metabolism to the TIA biosynthetic pathway was induced by tryptophan, secologanin and MeJA. TIAs are formed from strictosidine, and the monoterpene precursor, secologanin, which is produced by the MVA pathway, regulates the activity of the MEP pathway [40]. Sesquiterpenes are formed predominantly via the MVA pathway, while hemiterpenes, monoterpenes and diterpenes are mostly formed from the MEP pathway [41]. The monoterpene content of (−)-β-pinene increases after MeJA treatment in *Pinus sylvestris* and *Picea abies* seedlings [42]. Similarly, MeJA induces the biosynthesis of artemisinin sesquiterpenes in *Artemisia annua* *andabietane diterpenes in* *Salvia sclarea* by up-regulating the expression of related genes [43,44]. Our experimental results showed that the levels of carvone (a monterpene), miltirone (a diterpene) and rishitin (a sesquiterpene) were elevated under Trp + S + MeJA treatment. Overall, the above phenomena indicated that the metabolic flux was mainly directed to the TIA biosynthetic pathway and the terpene biosynthetic pathway induced by tryptophan, secologanin and MeJA.

By comparing the putative alkaloid biosynthesis response to TIA precursors (tryptophan and secologanin) and MeJA treatment in *D. officinale* PLBs studied in this work, we confirmed that jasmonate-mediated signaling pathways have a vital effect on TIA biosynthesis regulation. However, some key intermediates in this biosynthetic pathway, such as secologanin, tryptamine and stictosidine, were not detected in our study. It may due to the untargeted approach for alkaloids or intermediates characterizing. In the future, it is necessary to fully characterize carapanaubine by targeted isolation, purification and NMR (or standard compound) identification. And it would be worthwhile to identify the functional genes or transcription factors involved in the TIA biosynthesis of *D. officinale* response to MeJA by transcriptomic and proteomic studies and provide important clues for exploring the molecular mechanism of TIA biosynthesis regulation by feeding with MeJA-independent signals.

## 4. Materials and Methods

### 4.1. Plant Materials and Growth Conditions

**Figure 9:**
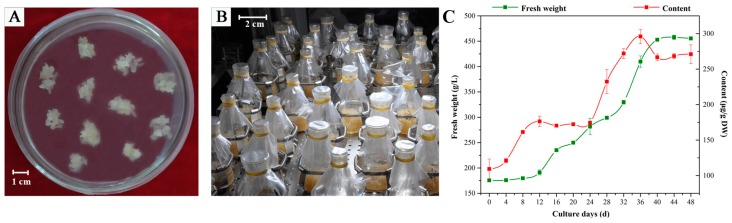
Liquid-suspension cultures of *D. officinale* protocorm-like bodies (PLBs). (**A**) PLB induction; (**B**) liquid-suspension cultures in 100 mL Erlenmeyer flasks and (**C**) cell growth and alkaloid accumulation curves during the whole culture period.

Protocorm-like bodies (PLBs) were induced from sterilized seeds of *D. officinale* (Figure 9A). In vitro-induced PLBs were maintained on 1/2 Murashige and Skoog (MS) liquid medium supplemented with 0.1 mg/L α-naphthalene acetic acid (NAA), 0.1 g/L lactalbumin hydrolysate and 30 g/L sucrose (pH of 5.8). The liquid-suspension cultures were kept in an incubator shaker set at a speed of 120 rpm in the dark at 25 °C and were routinely subcultured every 48 days (Figure 9B).

### 4.2. Chemicals and Reagents

Methanol, acetonitrile and chloroform (HPLC grade; ≥99.9%) were purchased from TEDIA (Fairfield, OH, USA). Pyridine was obtained from Ehrenstorfer (Augsburg, Germany). Dendrobine (HPLC grade; ≥98%) was purchased from Chengdu Mansite Bio-Technology Co., Ltd. (Chengdu, China). Adonitol (≥99%), methoxylamine hydrochloride (≥99%), tryptophan (≥98%) and secologanin (≥88%) were obtained from Sigma-Aldrich (St. Louis, MO, USA). *N*,*O*-bis (trimethylsilyl)-trifluoroacetamide (BSTFA) containing 1% trimethylchlorosilane (TMCS) was purchased from SUPELO (Bellefonte, PA, USA). Methyl jasmonate (≥95%) was purchased from Aladdin Industrial Corporation (Shanghai, China). Tryptamine (≥98%) and strictosidine (≥98%) were purchased from Shanghai Yuanye Biotechnology Co., Ltd. (Shanghai, China).

### 4.3. Elicitation and Precursor Feeding Experiments

Stock solutions of tryptophan, secologanin and MeJA were prepared. Tryptophan and secologanin were dissolved in water, and MeJA was dissolved in ethanol; all solutions were filter-sterilized through a 0.22 µm nylon filter. PLBs whose fresh weight was 7 g were inoculated into 40 mL of liquid culture medium in a 100 mL Erlenmeyer flask to select precursors and MeJA combined concentrations for obtaining the highest total alkaloid content. Tryptophan and secologanin were used as exogenous precursors at final concentrations of 3, 6 and 9 µM, whereas MeJA was used as the elicitor at final concentrations of 100, 200 and 300 µM. To determine overall effects of these 3 factors, the total alkaloid content was taken as indicator and orthogonal design of this experiment is shown in Table 3. A control group without tryptophan, secologanin and MeJA was also prepared. All results were based on six biological repeats, with three analytical replicates for each biological repeat. PLBs were harvested after 12, 24, 36 and 48 days based on the curves of fresh weight growth and alkaloid accumulation (Figure 9C).

### 4.4. Determination of Biomass and Total Alkaloid Content

The harvested PLBs were washed with running water. Fresh weight (FW, g/L) was measured after blotting the surface water with filter paper, and the dry weight (DW, g/L) was measured after drying at 60 °C for two days.

The method was slightly modified to analyze the total alkaloid content [14]. Briefly, 0.5 g of dried fresh PLBs was suspended in an ammonia solution for 0.5 h and then extracted with 25 mL of chloroform in a 50 mL conical flask. The flask was placed into a 70 °C water bath for 3 h, after which the chloroform was dried with a rotary evaporator. Afterward, 5 mL of chloroform was added to redissolve the dried residue, and 2 mL of the chloroform extract was mixed with 8 mL of chloroform. Five milliliters of potassium biphthalate buffer (pH 4.5) and 2 mL of 0.04% bromocresol green solution (*w*/*v*) were added. The mixture was left to stand for 30 min after intense shaking for 3 min. Subsequently, 1 mL of 0.01 mol/L NaOH (dissolved with ethanol) was added to 5 mL of the lower fractions for analysis. The total alkaloid content was determined by using a spectrophotometer at 620 nm, with dendrobine as the reference standard. The standard curve equation was *y* = 9.7829*x* + 0.0044 (*R*^2^ = 0.994), where *y* represents the absorbance of dendrobine and where *x* represents the dendrobine content (mg). The total alkaloid content was calculated using the following equation: total alkaloid content (μg/g DW) = dendrobine (mg) × 5 × 1000/0.5 g. All the results were presented as mean ± SD, statistical significance was determined by one-way analysis of variance (ANOVA) followed by the Duncan’s multiple range test (MRT) at a probability level of 95% (*p* = 0.05) [45].

### 4.5. Strictosidine Synthase Assay

Strictosidine synthase (STR) activity was measured using a modified protocol of a previous study [46,47]. One gram of PLBs was frozen in liquid nitrogen and ground to a fine powder. Fifty milligrams of polyvinylpolypyrrolidone and 1 mL of extraction buffer (0.1 mM phosphate buffer (pH 6.3), 3 mM ethylenediaminetetraacetic acid (EDTA) and 6 mM dithiothreitol (DTT)) were added. The mixture was then centrifuged at 18,000 rpm for 15 min at 4 °C. The supernatants were collected as protein extracts and stored at −20 °C. Fifty microliters protein extracts were incubated in 100 mM phosphate buffer (pH 6.8) that contained 0.8 mM tryptamine, 2 mM secologanin and 100 mM d-(+)-gluconic acid-δ-lactone (final reaction volume of 200 μL) for 30 min at 35 °C. The reaction was terminated by adding 100 μL of chilled methanol and filtered through a 0.22 µm nylon filter for HPLC analysis. STR enzymatic activity was determined indirectly by the quantity of strictosidine (µg/mL) in the reaction mixture. Strictosidine was analyzed using an Ultimate 3000 (Waters, Miford, MA, USA) HPLC system coupled to a Hypersil Gold column (4.6 mm × 250 mm × 5 μm, Thermo, Waltham, MA, USA). An aliquot of the reaction (10 μL) was directly injected using a solvent gradient of 15% acetonitrile in 0.05% formic acid for 20 min. The absorbance of strictosidine was monitored at 280 nm. 

### 4.6. Metabolite Extraction for GC-MS Analysis

The protocol for GC-MS metabolite extraction was in accordance with that of the reference [48], with slight changes. In brief, 10 mg of dried *D. officinale* PLB samples was transferred to a 2 mL tube and homogenized with a ball mill (ground for 2 min at a vibration frequency of 70 Hz). Metabolites were extracted with 1.4 mL of methanol (pre-chilled to −20 °C) and 60 µL of ribitol (0.2 mg/mL aqueous solution) as an internal standard, followed by vortexing for 10 s. The mixture was extracted using a supersonic instrument for 30 min (40 °C), and the tubes were centrifuged at 11,000 rpm for 10 min (4 °C). Subsequently, 1.2 mL of the supernatant was transferred to a fresh 10 mL tube, and 750 µL of chloroform with 1.4 mL of chilled water (4 °C) were added, followed by vortexing for 10 s. One hundred fifty microliters of the supernatant (polar phase) was collected in a derivatized glass bottle and dried under a nitrogen gas stream. The dried residue was re-dissolved in 80 µL of methoxylamine hydrochloride (20 mg/mL in pyridine), vortexed for 1 min, and incubated at 37 °C for 1.5 h. Then, 80 μL of BSTFA (containing 1% TMCS) was added to the mixture, which was then vortexed for 1 min, and heated at 70 °C for 1 h. Prior to injection, the solution was centrifuged at 11,000 rpm for 3 min (4 °C), and the supernatant was transferred to a glass vial for GC-MS analysis. To evaluate the reproducibility of GC-MS during the analysis, quality control samples (QCs) were prepared by mixing equal volumes (10 μL) of the all samples to be a pooled sample and processed identical to the study samples. The QCs were injected at regular intervals (every 4 study samples) throughout the analytical run to provide a set of data from which repeatability can be assessed. 

### 4.7. Metabolite Extraction for LC-MS Analysis

The alkaloid extraction method was performed as described by Akhgari [49], with slight modifications. Dried PLBs were accurately weighted twice to extract 50 mg using sonication for 30 min at 25 °C with methanol (2 × 1 mL). The mixture was then centrifuged at 11,000 rpm for 10 min, after which the supernatants (methanol extracts) were combined and evaporated to dryness under a nitrogen stream. The dried residue was re-dissolved with 2 mL of 10% sulfuric acid (*v*/*v*), after which the solution was then basified to pH 10 with 25% ammonia solution and vortexed. Afterward, the solution was incubated for 30 min at room temperature. This mixture was extracted twice with dichloromethane (2 × 1 mL), vortexed and then centrifuged at 4000 rpm for 15 min. The dichloromethane fractions were combined, evaporated to dryness, dissolved in 600 µL of methanol and then filtered through a 0.22 µm nylon filter prior to LC-MS analysis. QCs were also prepared to investigate the repeatability and stability of LC-MS, and carnitine C2:0-d3, carnitine C8:0-d3, carnitine C10:0-d3, carnitine C16:0-d3, LPC 19:0, FFA C16:0-d3, FFA C18:0-d3, CDCA-d4, CA-d4, Trp-d5, Phe-d5, SM12:0 and choline-d4 were added to the QCs for retention time calibration. The injection sequence was the same as for GC-MS.

### 4.8. Instrument Parameters for GC-MS

The GC-MS data were obtained using an Agilent 7890B-5977B (Agilent, Santa Clara, CA, USA) system equipped with an HP-5 MS capillary column (30 m × 0.25 mm, 0.25 μm film thickness, Agilent J & W Scientific, Folsom, CA, USA). Helium was used as a carrier gas at a constant flow rate of 1.0 mL/min. Samples of 1 μL were injected in split mode at a ratio of 10:1 and at a constant temperature of 280 °C. The ion source and interface temperatures were set to 230 and 250 °C, respectively. The initial temperature of the column was 40 °C for 5 min, after which it increased to 280 °C at a rate of 10 °C/min and remained constant for 5 min. Mass spectra were taken at 70 eV, and the mass range was from 35 to 780 *m*/*z*. The solvent delay was 8.5 min. 

### 4.9. Instrument Parameters for LC-MS

An Acquity Ultimate 3000 UHPLC system coupled to an LTQ Orbitrap MS (Thermo Fisher Scientific, Waltham, MA, USA) was used to analyze the metabolic profiling in both ESI positive and ESI negative ion modes. In the positive ion mode, the separation of metabolites was conducted on an Acquity TM BEH C8 column (1.7 μm, 2.1 × 100 mm, Waters, Milford, MA, USA), and the mobile phase contained water with 0.1% formic acid (A) and acetonitrile (B). The linear elution gradient program used as follows: 5% B for 1.0 min, a linear increase to 100% B at 24 min, holding for 4 min, decrease to 5% from 28 to 28.1 min, and holding at 5% from 28.1 to 30 min. In the negative ion mode, metabolite separation was performed using an Acquity TM HSS T3 column (1.8 μm, 2.1 × 100 mm, Waters, Milford, MA, USA). The mobile phase consisted of a 6.5 mM ammonium bicarbonate water solution (C) and 6.5 mM ammonium bicarbonate in 95% methanol and water (D). The linear elution gradient program was as follows: 5% D for 1.0 min, a linear increase to 100% D at 18 min, holding for 4 min, decrease to 5% from 22 to 22.1 min, and holding at 5% from 22.1 to 25 min. The flow rate was 0.35 mL/min, and the column temperature was 50 °C. The injection volume was 5 μL. Mass spectrometry detections were set as the following: capillary temperatures of 350 and 360 °C as well as spray voltages of 3.5 and 3.0 kV for positive ion mode and negative ion mode, respectively. Helium was used as the auxiliary gas with a flow rate of 30 and 40 arbitrary units for positive ion mode and negative ion mode, respectively. The mass scan range was 50 to 1000 *m*/*z*, and the resolution of the MS was set to 30,000. The collision-induced dissociation was set to normalized collision energy of 30%. For identification purposes, two scan modes were applied for the MS experiments. The first was in a full scan MS mode and the second was a data dependent scan that selected five most intense ions from the first scan event for the acquisition of MS/MS spectra.

### 4.10. Data Analysis

To assess the biological variance, six biological replicates of each *D. officinale* PLB were extracted and analyzed in parallel under identical conditions. The QCs were injected at regular intervals (every four samples) throughout the GC-MS and LC-MS analytical run to provide a set of data from which repeatability could be assessed. All the GC-MS and LC-MS data were processed by XCMS (Available online: http://www.bioconductor.org) running under the R package (Available online: http://www.r-project.org), which produced a matrix of features with the associated retention time, accurate mass and peak area. The variables presented in least 80% of either group were extracted. In addition, the variables with <30% relative standard deviation (RSD) in the QCs were then retained for further multivariate data analysis. All internal peaks were removed from the data set. The resulting data were normalized to the total peak area of each sample in Excel 2007 (Microsoft, Redmond, WA, USA). Data were imported into a Simca-P software version 14.0 (Umetrics AB, Umea, Sweden, Available online: www.umetrics.com/simca), where principal component analysis (PCA) and orthogonal partial least squares-discriminant analysis (OPLS-DA) were performed. The data scaling modes of PCA and OPLS-DA analyses were unit variance scaling and Pareto scaling, respectively. The quality of the models was described by the R^2^X or R^2^Y and Q^2^ values. The differential metabolites were selected on the basis of the combination of the statistically significant threshold of variable influence on projection (VIP) values obtained from the OPLS-DA model and the *p*-value from a two-tailed Student’s *t*-test of the normalized peak area; metabolites with VIP >1.5 and *p*-value < 0.05, respectively, were selected.

For GC-MS analysis, metabolites were identified by searching the commercial database NIST 11 [50] after their mass spectra were deconvoluted by the Automated Mass Spectral Deconvolution and Identification System (AMDIS) [51]. This spectral comparison provided putative empirical formulae and structures that were further searched in databases such as the Dictionary of Natural Products (Available online: http://www.Dnp.chemnetbase.com) and Chemspider (Available online: http://www.chemspider.com) [52,53]. Peaks with a similarity index of more than 80% were tentatively identified as metabolites. For LC-MS analysis, we used the software One-step Solution for Identification of Small Molecules in Metabolomics Studies (OSI/SMMS), which was co-developed by the Dalian Institute of Chemical Physics, Chinese Academy of Sciences and Dalian ChemData Solution Information Technology Co., Ltd. (Dalian, China), to identify the metabolites. A reference material database (in-house mass spectral library), HMDB (Available online: http://www.hmdb.ca/) and METLIN (Available online: http://metlin.scripps.edu/) were used. The identified metabolites were mapped to general biochemical pathways according to their annotation in the Kyoto Encyclopedia of Genes and Genomes (KEGG) database (Available online: http://www.kegg.jp/kegg/pathway.html). Pathway analysis was performed using MetaboAnalyst 3.0 (Available online: http://www.metaboanalyst.ca/MetaboAnalyst/) [54]. The metabolome data of the differential metabolites were used for this analysis, and subsequently normalized by sum, log transformed, Pareto scaled and compared with the KEGG pathway libarary of *Oryza sativa* japonica and *Arabidopsis thaliana.* Metabolite-metabolite correlations between identified metabolites were analyzed by using the Pearson correlation method in R software. The metabolic network based on the results of the Pearson correlation analysis was constructed by Cytoscape software version 2.8.3 (Cytoscape Consortium, San Diego, CA, USA, Available online: www.cytoscape.org/). The heatmap generation was also performed with R software.

## 5. Conclusions

This study traced the metabolic profiling of alkaloid biosynthesis in *D. officinale* in response to TIA precursors (tryptophan and secologanin) and MeJA treatment. It was observed that the total alkaloid content increased most noticeably when the PLBs were fed with 9 µM tryptophan, 6 µM secologanin and 100 µM MeJA after 36 days. A total of 78 types of metabolites were identified by untargeted metabolomics methods using GC-MS in combination with LC-MS, and 16 of those metabolites were alkaloids, which were classified into eight categories: TIAs, tropane alkaloids, monoterpene alkaloids, diterpene alkaloids, pyrrolizidine alkaloids, isoquinoline alkaloids, quinolizidine alkaloids and piperidine alkaloids. Twenty nine metabolites were identified (VIP > 1.5 and *p* < 0.05) as differential metabolites. The level of carapanaubine (a kind of TIA) dramatically increased under this combinative treatment, and a possible underlying process of the metabolic flux from related metabolism (sugar, pyrrolizidine alkaloid and tropane alkaloid metabolism) to the TIA biosynthetic pathway (including the terpene biosynthetic pathway) was also enhanced. To conclude, our findings support the hypothesis that *D. officinale* contained terpenoid indole alkaloid and the biosynthetic pathway was affected by TIA precursors and MeJA treatment.

## Figures and Tables

**Table 1:** Orthogonal array of experiments on the total alkaloid content of *D. officinale* PLBs.

No.	[A] Tryptophan (µM)	[B] Secologanin (µM)	[C] MeJA (µM)	Total Alkaloid Content (μg/g DW)
1	1 (3)	1 (3)	1 (100)	188
2	1	2 (6)	2 (200)	135
3	1	3 (9)	3 (300)	190
4	2 (6)	1	2	190
5	2	2	3	190
6	2	3	1	257
7	3 (9)	1	3	124
8	3	2	1	472
9	3	3	2	177
*K*_1_	171.000	167.667	305.667	
*K*_2_	212.333	265.667	167.333	
*K*_3_	258.000	208.000	168.333	
Range	87.000	98.000	138.334	
Order	C > B > A			
Optimal level	A3	B2	C1	
Optimal group	A3B2C1			

The numbers in brackets represent the concentrations of three factors. PLBs: protocorm-like bodies; MeJA: methyl jasmonate; DW: dry weight.

**Table 2:** Differential metabolites identified from the OPLS-DA and correlated with the TIA precursors and MeJA treatment in *D. officinale* PLBs.

No.	Metabolites	Catogery	VIP	Change Fold (Trp + S + MeJA/Control)	*p*-Value
1	Tryptophan	Amino acid	4.57	20.65	** 7.43 × 10^−15^
2	Glycine	Amino acid	3.82	20.65	** 7.43 × 10^−15^
3	Aspartic acid	Amino acid	4.36	0.59	** 1.79 × 10^−9^
4	Glutamic acid	Amino acid	2.18	0.54	* 0.03
5	Fructose	Sugar	6.29	0.31	** 5.15 × 10^−12^
6	Trehalose	Sugar	1.51	3.03	** 5.98 × 10^−13^
7	Glucose	Sugar	8.97	0.51	** 4.23 × 10^−10^
8	Mannose	Sugar	4.92	0.72	** 9.22 × 10^−8^
9	Galactose	Sugar	8.41	0.47	** 9.78 × 10^−11^
10	Arabinose	Sugar	3.27	2.69	** 1.43 × 10^−12^
11	Stearic acid	Fatty acid	11.53	1.13	* 0.02
12	Arachidonic acid	Fatty acid	5.14	0.07	** 1.29 × 10^−7^
13	Eicosanoic acid	Fatty acid	6.06	1.47	** 1.00 × 10^−7^
14	Linolenic acid	Fatty acid	10.68	1.45	** 0.003
15	Pentadecanoic acid	Fatty acid	2.45	0.83	** 0.005
16	Glycerol	Alcohol	5.27	0.65	** 5.61 × 10^−9^
17	Myo-inositol	Alcohol	3.79	0.77	** 7.17 × 10^−7^
18	Malic acid	Organic acid	2.64	0.46	** 0.005
19	Shikimic acid	Organic acid	8.61	0.78	** 9.78 × 10^−11^
20	Carapanaubine	Terpenoid indole alkaloid	2.98	1928.46	** 1.77 × 10^−7^
21	Pelletierine	Piperidine alkaloid	5.38	0.57	** 3.61 × 10^−5^
22	Senkirkine	Pyrrolizidine alkaloid	3.69	0.68	** 0.001
23	Xanthoplanine	Isoquinoline alkaloid	3.34	1.13	* 0.018
24	Carvone	Terpene	3.17	8.81	** 3.12 × 10^−12^
25	Rishitin	Terpene	2.45	26.74	** 4.88 × 10^−13^
26	Miltirone	Terpene	4.53	0.41	** 4.25 × 10^−6^
27	Indole-3-ethanol	Other	3.22	2.18	** 1.00 × 10^−7^
28	Piperidine	Other	1.61	7.24	** 2.37 × 10^−14^
29	Camelliagenin C	Saponin	3.23	0.58	* 0.013

Significant * *p* < 0.05, extremely significant ** *p* < 0.01.

**Table 3:** Orthogonal experimental design (three factors and three levels).

Level	Factors		
	[A] Tryptophan (µM)	[B] Secologanin (µM)	[C] MeJA (µM)
1	3	3	100
2	6	6	200
3	9	9	300

## Supplementary Materials

Supplementary materials can be found at http://www.mdpi.com/1422-0067/19/3/728/s1.
