# Supplementary material for: Metabolic Profiling of Dendrobium officinale in Response to Precursors and Methyl Jasmonate
Source: Int J Mol Sci. 2018 Mar 3;19(3):728. doi: 10.3390/ijms19030728 (PMC5877589; doi:10.3390/ijms19030728)
Supplement: Supplementary file 1 [file ijms-19-00728-s001.zip › ijms-271021 supplementary r2/Supplementary materials 1.doc]

**Supplementary Materials:
Metabolic Profiling of *Dendrobium officinale* in Response to Precursors and Methyl Jasmonate**

**Chunyan Jiao, Cheng Song, Siyan Zheng, Yingpeng Zhu, Qing Jin, Yongping Cai and Yi Lin**

**Figure S1.** LC-MS spectra of extracts from treated (9 µM tryptophan, 6 µM secologanin and 100 µM MeJA incubated for 36 days) and untreated *D. officinale* PLBs.

**Figure S2.** Permutation test for the OPLS-DA model. The permutation plot displays the correlation coefficient between the original *y*-variable and the permuted *y*-variable on the *X*-axis versus the cumulative R^2^ and Q^2^ on the *Y*-axis, and the regression line was drawn. The intercept is a measure of the overfit.

**Table S1.** Effects of TIA precursors and MeJA on the fresh weight (FW, g/L) of *D. officinale* PLBs during different culture days by an orthogonal layout.

| **No.** | **Culture days (d)** | | | |
| --- | --- | --- | --- | --- |
|  | **12** | **24** | **36** | **48** |
| Control (0) | 191.16±4.98 a | 281.68±16.17 a | 409.77±11.18 a | 455.58±3.43 a |
| 1 | 172.21±0.78 cd | 227.27±3.75 b | 373.36±7.85 b | 405.34±5.09 c |
| 2 | 174.02±1.22 c | 206.18±2.68 c | 297.99±2.59 d | 319.20±2.91 f |
| 3 | 164.23±0.48 f | 182.54±3.12 e | 246.78±1.94 f | 246.23±1.27 i |
| 4 | 168.53±0.86 cde | 209.23±2.01 c | 314.30±1.66 c | 370.37±13.94 e |
| 5 | 165.16±1.88 f | 193.45±3.43 d | 266.20±1.46 e | 280.17±4.41 h |
| 6 | 180.34±1.41 b | 229.15±4.46 b | 371.73±5.52 b | 401.58±4.24 c |
| 7 | 166.52±1.44 de | 195.24±2.00 d | 223.37±1.56 g | 306.63±2.06 g |
| 8 | 180.10±8.38 b | 235.74±2.31 b | 359.53±22.08 b | 416.58±1.64 b |
| 9 | 172.47±1.91 cd | 208.32±0.85 c | 296.43±4.70 d | 391.18±2.31 d |

Data are means of six replication. Mean values followed by the same letters are not significantly different (*p*=0.05) according to Duncan’s multiple range test.

**Table S2.** Effects of TIA precursors and MeJA on the dry weight (DW, g/L) of D. officinale PLBs during different culture days by an orthogonal layout.

| **No.** | **Culture days (d)** | | | |
| --- | --- | --- | --- | --- |
|  | **12** | **24** | **36** | **48** |
| Control (0) | 21.89±1.08 ab | 26.36±1.61 a | 24.33±1.80 bc | 21.59±0.23 b |
| 1 | 21.00±0.18 c | 26.03±1.64 ab | 23.26±0.36 cd | 21.50±0.15 b |
| 2 | 20.24±0.38 d | 24.35±2.10 ab | 26.05±1.26 a | 21.42±0.26 b |
| 3 | 17.93±0.35 f | 23.15±0.25 b | 23.13±0.18 cd | 21.95±0.09 a |
| 4 | 19.28±0.15 e | 26.23±1.86 a | 23.18±0.20 cd | 21.28±0.12 b |
| 5 | 18.98±0.05 e | 24.54±0.15 ab | 24.68±0.12 b | 20.67±0.19 cd |
| 6 | 21.25±0.16 bc | 27.08±1.74 a | 22.78±0.13 d | 19.50±0.13 e |
| 7 | 18.12±0.15 f | 24.85±0.71 ab | 24.24±0.35 bc | 20.38±0.25 d |
| 8 | 22.24±0.08 a | 26.15±2.39 a | 20.53±0.33 e | 20.82±0.14 c |
| 9 | 21.09±0.34 c | 25.38±0.84 ab | 24.78±0.11 b | 18.94±0.24 f |

Data are means of six replication. Mean values followed by the same letters are not significantly different (*p*=0.05) according to Duncan’s multiple range test.

**Table S3.** Effects of TIA precursors and MeJA on the total alkaloid content (µg/g DW) of *D. officinale* PLBs during different culture days by an orthogonal layout.

| **No.** | **Culture days (d)** | | | |
| --- | --- | --- | --- | --- |
|  | **12** | **24** | **36** | **48** |
| Control (0) | 162.67±26.41 d | 162.67±9.50 a | 230.00±10.15 b | 271.00±13.23 ef |
| 1 | 211.33±11.72 a | 108.67±3.06 c | 188.33±2.52 d | 369.33±14.19 b |
| 2 | 105.33±5.51 e | 99.03±2.44 c | 135.33±7.02 e | 216.00±8.54 h |
| 3 | 100.33±5.51 e | 89.57±7.91 d | 190.00±20.00 d | 251.00±4.00 fg |
| 4 | 94.67±9.98 e | 157.00±6.56 a | 190.00±17.32 d | 305.67±9.02 d |
| 5 | 177.00±6.56 cd | 129.00±5.00 b | 190.00±10.00 d | 292.33±6.66 de |
| 6 | 188.33±4.04 bc | 96.00±6.24 d | 257.33±4.04 c | 347.00±5.00 c |
| 7 | 196.00±6.24 ab | 89.00±7.00 d | 124.37±3.06 c | 238.33±11.24 g |
| 8 | 105.67±1.53 e | 123.37±6.11 b | 472.33±3.79 a | 425.67±28.01 a |
| 9 | 172.67±5.69 cd | 90.83±6.58 d | 176.67±15.28 d | 390.67±7.23 b |

Data are means of six replication. Mean values followed by the same letters are not significantly different (*p*=0.05) according to Duncan’s multiple range test.

**Table S4.** Variance analysis of orthogonal experiment.

| **Variance** | **SS** | **df** | ***F* value** | **Significance** |
| --- | --- | --- | --- | --- |
| A | 11274.667 | 2 | 0.501 | - |
| B | 14648.667 | 2 | 0.651 | - |
| C | 38088.667 | 2 | 1.692 | - |

SS: Sum of squares; df: Degree of freedom.

**Table S5.** Results of the validation test.

| **No.** | **Total alkaloid content (μg/g DW）** |
| --- | --- |
| 1 | 474 |
| 2 | 470 |
| 3 | 475 |
| Mean value | 473 |
